# Women and ARV-based HIV prevention – challenges and opportunities

**DOI:** 10.7448/IAS.17.3.19356

**Published:** 2014-09-08

**Authors:** Cynthia W Geary, Elizabeth A Bukusi

**Affiliations:** 1 FHI 360, Durham, NC, USA; 2 Centre for Microbiology Research, Kenya Medical Research Institute, Nairobi, Kenya; 3 Departments of Obstetrics and Gynecology, and Global health, University of Washington, Seattle,WA, USA; 4 Department of Obstetrics and Gynecology Aga Khan University, Nairobi, Kenya; 5 Department of Obstetrics and Gynecology, Nairobi University, Nairobi, Kenya; 6 Department of Obstetrics, Gynecology and Reproductive Sciences, University of California San Francisco, USA

**Keywords:** women, gender, HIV prevention, ARVs, microbicides, PrEP, risk compensation

## Abstract

ARV-based HIV prevention methods available in pill, gel or ring formulations (broadly referred to as microbicides) offer the possibility of protection against HIV for women who find it difficult because they cannot ask their partners to use condoms or even refuse sex. Partial efficacy of ARV-based medications has been demonstrated in a number of clinical trials around the world among various populations, building the evidence that ARV-based technologies will contribute to reducing the AIDS epidemic worldwide. Disappointing results, however, from two trials in sub-Saharan Africa, where poor adherence contributed to study closure due to futility, have raised questions about whether women at the centre of the epidemic are able to effectively use products that require routine use. Also, there are fears by some of risk compensation by decreased condom use because of the availability of microbicides when only partial efficacy has been demonstrated in microbicide trials to date. Of note, sub-analyses of biologic measures of adherence in trials where this was possible have shown a strong correlation between good adherence and efficacy, reinforcing the necessity of good adherence. Research conducted in conjunction with clinical trials and post-trials in advance of possible rollout of ARV-based products have examined social and cultural factors, gender-related and otherwise, influencing adherence and other aspects of women's use of products. These include HIV stigma, women's perception of risk, partner and community influences and the differing needs of women in various stages of life and in different circumstances. It is the purpose of this supplement to give voice to the needs of women who can benefit from woman-initiated methods by presenting research results and commentary to contribute to the global conversation about optimizing women's experience with ARV-based prevention.

## Introduction

Many women around the world find it difficult to protect themselves against HIV infection because traditional gender norms surrounding sexual relationships and practice make it difficult to say ‘no’ to sex and difficult to ask that their partners use condoms. A request to use condoms can be interpreted as a sign of infidelity and lead to abandonment or violence [1]. For women in these circumstances, an HIV prevention method which they can use without their partners’ permission or knowledge, offers greater possibilities for protection than do exhortations to abstain or use condoms. The use of anti-retrovirals (ARVs), commonly used to treat people living with HIV (PLHIV), in gel, pill or ring formulations offers the possibility of greater agency for women to protect themselves. Partial efficacy of ARV-based prevention products has been demonstrated among several groups: heterosexual women [2] and men [3], MSM [4], sero-discordant couples [5], and drug injectors [6].

Reducing HIV transmission among women in sub-Saharan Africa is key to ending the global AIDS epidemic, where over 70% of all new infections occur each year. Out of the over 16 million women living with HIV globally, over 12 million are in sub-Saharan Africa [7]. Disappointingly, however, the initially promising results of ARV-based technologies to prevent HIV transmission were absent in two clinical trials with women in this part of the world: FEM-PrEP and VOICE which tested oral pre-exposure prophylaxis (PrEP) and microbicidal vaginal gel in women living in areas of high incidence. FEM-PrEP tested daily dosing of oral PrEP among women primarily in South Africa and Kenya [8], and VOICE evaluated two types of oral PrEP and a vaginal gel among women in South Africa, Uganda and Zimbabwe [9]. Adherence was so low in the FEM-PrEP and VOICE clinical trials that no determination could be made of the efficacy of the products. In the VOICE trial, less than a third of the women took the pills daily as prescribed. In the FEM-PrEP trial, good adherence (defined as having the amount of TFV in plasma estimated to be equivalent of a participant four or more times each week over the preceding 28 days) was found in only 12% of the sub-sample [10].

Because of expected challenges with adherence in these resource-poor settings, concerted effort was made to understand participant perceptions and address these challenges prior to and during these trials through formative and on-going qualitative research and adherence support counselling [8, 9]. The extremely low adherence rates found in spite of these efforts was a surprise and disappointment to researchers. Even with community engagement and education, results of qualitative research with trial participants during and following these trials has found that many rumours and misperceptions remained throughout the studies, indicating the strong influence of cultural and social understandings and norms on emotionally charged issues [11, 12].

The expressed concern about women's lack of use of microbicides has emphasized adherence, but in reality this has just become shorthand for describing a number of issues that prevent women's effective use of these products. Though many women who chose to enrol in the clinical trials were able to use gel or PrEP effectively, there were often social and cultural barriers, gender-related and otherwise, that made it difficult for others. Based on data collected from women who were trial participants prior to and during clinical trials, we know that some of these potentially addressable issues for women's use of ARV based prevention included HIV stigma, women's lack of understanding and misperceptions of how these products protect them against HIV, misperceptions of their HIV risk as well as negative perceptions of an investigational drug and lack of support by their partners and the wider community [11–13].

A tension that remains in the discussion of the appropriateness of microbicides for women relates to the partial efficacy of microbicides found to date and the fear of risk compensation reducing the role of condoms in HIV prevention [14]. To date, all studies that have been able to measure efficacy have shown only partial efficacy [15], and so for optimal effectiveness, and to prevent other STIs, microbicides should be used in tandem with condoms, if possible. This can be a big ‘if,’ however. As noted, the initial motivation for a female initiated product was that many women live in circumstances they cannot control and they cannot ask a partner to use a condom without fear of rejection or violence. Optimally, microbicides should be introduced into a service delivery setting in conjunction with larger efforts to change social and cultural norms and involve men in supporting women's microbicide use if possible [16], but changing norms is a long-term solution. Many women – especially in places where there is high HIV incidence – need the best solution they can find now. The fear that the availability of microbicides will reduce men's motivation to use a condom is relevant and real in places where it is normative for men to use condoms, but an examination of DHS data on men's condom use in the 10 countries with the highest HIV prevalence finds in most of these countries significantly less than half report using a condom at last sex with any partner [17]. Sub-analyses of FEM-PrEP and VOICE trial data indicated that greater adherence to the prescribed dosing regimen – measured through blood drug levels – correlated with greater product efficacy [9], an indication that efficacy estimates will likely improve if adherence improves.

Dramatically improved adherence to these ARV-based products is imperative if they are to be effective in reducing transmission in this population critical to ending the global epidemic. The results on adherence from the VOICE and FEM-PrEP trials have led people in the field to express concerns about whether a female initiated technology that requires routine use is a viable option for women at all [18]. One strategy to address this concern has been to focus efforts on methods for women that are less user-dependent, but a more effective strategy that recognizes the differing needs of women in different situations may be to increase the options that women have to protect themselves against HIV infection.

It is the purpose of the papers in this supplement to give voice to the needs of women who can benefit from woman-initiated methods by presenting research, analysis, and commentary that contribute to the global conversation of how to minimize challenges and maximize opportunities for the effective use of ARV-based products by women. The supplement begins with a synthesis by Mastro *et al*. [15] of what is known about the efficacy of various ARV-based prevention methods in women. Social, cultural and policy issues related to microbicide development and potential rollout guidelines and policies are reflected in the remaining papers in this supplement – women's perception of risk, community influence on participants’ adherence, adherence support, understanding the needs and preferences of groups of end-users who are characterized by specific circumstances or at different stages in the life cycle, the tricky issue of partner involvement and potential roles for community, providers and the private sector in optimizing women's experience with ARV-based prevention.

## Perceptions of clinical trial participants

In exploring adherence, issues among participants in the VOICE trial, van der Straten *et al*. [11] found that the way community members conceptualize disease transmission and its treatment can influence participants’ behaviours in prevention trials. Use of ARVs was confusing to many community members and contributed to stigma experienced by participants and community mistrust.

Another proposed factor related to poor adherence is perception of low sexual risk. Among the 68 seroconverters in the FEM-PrEP trial by Corneli *et al*., half reported that they had no chance of becoming infected four weeks before they tested positive. Rationalizations for perceptions of HIV risk were examined in qualitative interviews among 51 of the seroconverters [13]. Some women were assured by engaging in preventive behaviours such as PrEP or condom use, while others made assumptions based on prior non-infection or trust in their partners. Some women did worry about infection but felt unable to protect themselves for a variety of reasons. Study findings point to the need for adherence counselling discussions of HIV risk tailored to the specific situations of trial participants.

## Increasing adherence

The development of a vaginal ring for microbicides delivery is thought by some to take care of the adherence ‘problem’ by requiring less user involvement. Clinical trials, however, indicate that perfect adherence to the vaginal ring is not automatic. A commentary by MacQueen *et al*. [19], presents a framework for considering the coordination of three inter-related types of research relevant to increasing adherence to vaginal rings in clinical trials. These include improved, theory-based adherence support interventions; validated, generalizable self-reported psychometric scales; and development of real-time adherence monitoring using ‘smart’ or biometric technologies to detect ring insertion and/or removal or expulsion. A case is made for the need to conduct these three activities in a coordinated, inter-disciplinary way, to allow for the best thinking from experts in multiple fields in order to take advantage of synergies that may arise from a common discussion of adherence through multiple lenses.

## Understanding needs of specific groups of end-users

Several papers in this supplement report on research targeting various groups of women for whom microbicides are thought to be appropriate in specific contexts. First, Tolley and her colleagues [20] make the case for including adolescent girls aged 15–17 in HIV prevention trials based on the results of a mixed methods study in Tanzania. She and her colleagues found girls in this age range to be at similar or higher risk of infection to their 18- to 21-year-old counterparts but perceiving themselves at lower risk and under-utilizing prevention services. Though some might argue that these facts alone do not warrant the IRB complications related to including minors in clinical research, the authors argue that this age group may not be targeted for ARV-based prevention services if they are not included in clinical trial research initially.

A second paper, by Sidibe *et al*. [21], describes potential barriers and facilitators of microbicide use by four end-user audiences in Kenya based on a literature review and an in-country policy consultation: female sex workers, women in stable and discordant relationships, and sexually active young women. These groups varied in the extent to which the salience of HIV prevention versus partner intimacy or sexual pleasure as a motivation for using a vaginal microbicidal gel might encourage or discourage their use of such a product. The authors make suggestions for tailoring messages to meet the various needs of women.

Mack and her colleagues [22] also compared the interest in pre-exposure prophylaxis and preferences among three possible formulations – pills, gels and injectables – among several groups of potential end-users of ARV-based prevention methods in Kenya (females sex workers and sero-discordant couples) and South Africa (adolescents and young women). Her findings also support the varying needs of different types of end-users and the importance of formulation choice to create and sustain demand of different users in various contexts.

## The role of men in women's microbicide use

The issue of how to engage male partners to support but not manipulate women's use of microbicides has been raised in many stakeholder conversations anticipating the eventual rollout of ARV-based products. Lanham *et al*. [16] identified opportunities for new retrospective data collection relevant to this question from male partners of female participants in the three studies in Kenya, as well as a wealth of qualitative data from various microbicide trials that until then had been unanalysed. Analyses of these new and old data were presented in a meeting in 2013 to look for answers to this question. The meeting elicited rich discussion and synthesized guidelines for optimizing supportive male participation while protecting against unwanted interference.

## Improving women's experience with ARV-based prevention

The use of microbicides and PrEP, as with any other medications, requires attention to effective communication with users and with members of the communities in which they live; it is within these communities that many people's beliefs about illness and sex are formed, as well as their beliefs about how medications may or may not prevent illness. The use of vaginal products during sexual intercourse, for example, may be a novel practice requiring community-approved messages. Woodsong *et al*. [23] describes a process used for involving a community advisory board to ensure local comprehension of informational materials for women. These materials used visual images because of the low literacy of the study materials, but visual images also are subject to cultural interpretation. As such, comprehension of these materials was improved by the advice of local stakeholders.

Another paper by Lin *et al*. [24] considers what can be learned from the private sector about understanding the needs of the end-users, in this case, women, when introducing new medical products such as microbicides. One framework offered is that of the ‘user journey’ that takes into account various aspects of women's lives that might play into decisions relevant to the use of microbicides, with the objective of creating an interface between user and product that enhances women's likelihood of effective use.

WHO guidelines play a critical role in the introduction of new products within national health ministries. The last paper in the supplement by Lusti-Narasimhan [25] considers the relevance of gender norms in developing guidelines for microbicide or PrEP services. The proactive consideration of the influence of gender norms in the use of ARV-based technologies can help policymakers and programme managers facilitate women's full access to their use by considering issues such as protection of privacy and confidentiality in service delivery and reducing barriers such as parental consent that may keep adolescents at risk from using them.

## Conclusion

The availability of any biomedical technology does not obviate the need for consumer involvement in the correct and consistent use of that technology. Likewise, the development of a ‘woman-initiated’ technology does not really give a woman control until all the barriers to the use of that technology stemming from myriad social and gender inequities are removed. The promise of ARV-based technologies to empower women to protect themselves against HIV will be kept only to the extent that these inequities are considered and addressed in the development and implementation of gender transformative policy and service delivery models.
